# Editorial: Organellar dynamics in cell fate

**DOI:** 10.3389/fmolb.2022.977014

**Published:** 2022-08-05

**Authors:** Yuzhen Li, Xianglan Yao, Stewart J. Levine, Sang-Bing Ong

**Affiliations:** ^1^ Department of Basic Medicine, Graduate School of PLA General Hospital, Beijing, China; ^2^ Laboratory of Asthma and Lung Inflammation, Pulmonary Branch, National Heart, Lung and Blood Institute, National Institutes of Health, Bethesda, MD, United States; ^3^ Department of Medicine and Therapeutics, Faculty of Medicine, Chinese University of Hong Kong (CUHK), Hong Kong, Hong Kong SAR, China; ^4^ Centre for Cardiovascular Genomics and Medicine (CCGM), Lui Che Woo Institute of Innovative Medicine, Chinese University of Hong Kong (CUHK), Hong Kong, Hong Kong SAR, China; ^5^ Hong Kong Hub of Paediatric Excellence (HK HOPE), Hong Kong Children’s Hospital (HKCH), Kowloon Bay, Hong Kong SAR, China; ^6^ Kunming Institute of Zoology—The Chinese University of Hong Kong (KIZ-CUHK) Joint Laboratory of Bioresources and Molecular Research of Common Diseases, Kunming Institute of Zoology, Chinese Academy of Sciences, Kunming, China

**Keywords:** organellar dynamics, cardiac differentiation, non-coding RNA, endoplasmic reticulum, mitochondria, cardiomyocyte fate, MFN2, necroptosis

One of the intriguing aspects of cell fate lies in the fact that multiple factors, or a myriad of interconnecting processes dictates life and death of the cell. Among the many different triggers of death versus promoters of survival, many originate from the inside of the cell itself, with organelles being the upstream source or acting as a downstream converging interacting point for these factors. The organelles constitute the sites of crucial processes to maintain life such as replication/proliferation, differentiation, metabolism and energy generation while also mediating cell death via processes ranging from disrupted calcium homeostasis to release of pro-death proteins. Although investigation is ongoing, there remains unknown facets to the role of the organelles in governing cell fate. In this Research Topic comprising of 3 original research articles and 2 reviews, we get a glimpse of some of the different studies focused on unraveling the mysteries regarding the organelles as gatekeepers of life and death of the host cell.

Dai et al. from the group of Jian Wu and Yunzeng Zou demonstrated that cardiac hypertrophic preconditioning (HP) in mice preserved cardiac function, alleviated myocardial hypertrophy and fibrosis, provided that caspase-1 as a key member of the proinflammatory caspases known to mediate mitochondrial disassembly and its downstream cytokine targets IL-1β and IL-18 remain subdued. The authors found that HP abrogated the function of caspase-1 through attenuation of inflammation rather than pyroptosis. Genetic overexpression of caspase-1 in the myocardium prevented the benefit of HP in pressure overload mouse hearts via an aggravated activation of IL-1β and IL-18.

In another study by Tsoi et al., the authors investigated the optimal differentiation of hiPSCs to cardiomyocytes, from the perspective of Wnt signaling whereby repressing Wnt at the later stage is required to drive cardiac differentiation. The study found that the schedule of differentiation, rather than the choice of Wnt inhibitors, determined the cardiac differentiation and maturation efficiency of hiPSCs. The protocol employed in the study resulted in a significantly greater mitochondrial mass consistent with higher levels of mitochondrial genes detected by qPCR. The resulting hiPSC-CMs were also detected to have a switch to fatty acid oxidation with a higher spare respiratory capacity, signifying a high differentiation efficiency and enhanced developmental maturation. A Seahorse metabolic flux analysis revealed a significantly higher spare respiratory capacity in the hiPSC-derived cardiomyocytes using this modified algorithm of temporal control of Wnt signaling, alongside a significantly lower average frequency of cytosolic calcium (Ca^2+^) release, consistent of a more mature cardiomyocyte-like phenotype.

To further elaborate on the role of the pro-mitochondrial fusion protein -Mitofusin 2 (Mfn2) in mediating cardiac failure, Chen et al. from Gong Guohua’s team provided a multi-faceted dissection of how Mfn2 acts in a pleiotropic fashion to modulate mitochondrial fusion, contact and exchange with the ER for ER-mitochondria (Ca^2+^) regulation, autophagy and cellular apoptosis, culminating in cardiac hypertrophy and heart failure.

Alongside this, Yang et al. from Yaozu Xiang’s team aligned the role of the multifunctional serine/threonine protein kinase—Ca^2+^/calmodulin-dependent protein kinase II (CaMKII) in mediating IR-mediated cardiomyocyte death via intracellular mitochondrial swelling, and ER dysfunction in the form of Ca^2+^ leakage. The authors reflected on the effect of different posttranslational modifications of CaMKII in regulating myocardial I/R injury through the pro-inflammatory cascade, and cardiomyocyte death pathways including necroptosis and pyroptosis. In addition, the readers were also introduced to the CaMKII inhibitors that are currently in clinical trials for targeting different disorders as well as different drugs that may modify the CaMKII pathway and its related pathway in I/R.

One of the key regulators of cell fate that is under intense investigation currently is the non-coding RNAs consisting of the microRNA (miRNA) as well as long non-coding RNAs (lncRNAs). In a study investigating the role of lncRNA in mediating obesity, Xiao et al. demonstrated that the metabolism-sensitive lncRNA (named Lnc-FR332443) as the antisense lncRNA of Runx1 - a transcription factor that regulates differentiation of hematopoietic stem cells into mature blood cells, is highly enriched in adipose tissue and downregulated during adipogenic differentiation, with Runx1 mirroring it is lncRNA counterpart. Lnc-FR332443 positively regulates Runx1 expression in mouse adipocytes while suppressing adipocyte differentiation by attenuating the phosphorylation of MAPK-p38 and MAPK-ERK1/2 expression which are known to orchestrate cellular stress responses as well as cellular proliferation, differentiation, survival and migration. Overexpression of Runx1 downregulated the expression of the adipocyte cell marker genes PPARγ, C/EBPα and FABP4 significantly, without affecting the expression of Lnc-FR332443. In brief, the elucidation of the role of Lnc-FR332443 in the negative regulation of adipogenesis may suggest it is potential as a therapeutic target via epigenetic drug intervention against obesity and its related metabolic diseases.

In summary, this Research Topic highlights some of the current efforts in understanding how the changes in organelles, particularly the mitochondria and ER, are intertwined with physiological and pathophysiological processes to govern cell fate. Further investigations will enhance the elucidation of the role and relationship of inter-organellar dynamics in life and death of the cell, and potentially serve as therapeutic targets in combating against various disorders.

